# The Release of a Highly Cytotoxic Paullone Bearing a TEMPO Free Radical from the HSA Hydrogel: An EPR Spectroscopic Characterization

**DOI:** 10.3390/pharmaceutics14061174

**Published:** 2022-05-30

**Authors:** Ana Vesković, Đura Nakarada, Olga Vasiljević, Anatolie Dobrov, Gabriella Spengler, Éva A. Enyedy, Vladimir B. Arion, Ana Popović Bijelić

**Affiliations:** 1 Faculty of Physical Chemistry, University of Belgrade, Studentski trg 12-16, 11158 Belgrade, Serbia; ana.veskovic@ffh.bg.ac.rs (A.V.); djura@ffh.bg.ac.rs (Đ.N.); oglavasiljevic@gmail.com (O.V.); 2 Institute of Inorganic Chemistry, University of Vienna, Währinger Strasse 42, A-1090 Vienna, Austria; anatolie.dobrov@univie.ac.at (A.D.); vladimir.arion@univie.ac.at (V.B.A.); 3 MTA-SZTE Lendület Functional Metal Complexes Research Group, Department of Inorganic and Analytical Chemistry, University of Szeged, Dóm tér 7, H-6720 Szeged, Hungary; spengler.gabriella@med.u-szeged.hu (G.S.); enyedy@chem.u-szeged.hu (É.A.E.); 4 Department of Medical Microbiology, Albert Szent-Györgyi Health Center and Albert Szent-Györgyi Medical School, University of Szeged, Semmelweis u. 6, H-6725 Szeged, Hungary

**Keywords:** cytotoxic ligand, drug release, EPR spectroscopy and imaging, HSA hydrogel, paullones, spin labeling

## Abstract

This study shows the potential of a thermally induced human serum albumin (HSA) hydrogel to serve as a drug depot for sustained release of a highly cytotoxic modified paullone ligand bearing a TEMPO free radical (**HL**). The binding of **HL** to HSA was studied by electron paramagnetic resonance (EPR) spectroscopy and imaging. The EPR protocol was also implemented for the study of matrix degradation, and ligand diffusion rate, in two additional spin-labeled hydrogels, containing 5-doxylstearate and 3-carbamoyl-proxyl. The results showed that the hydrogel is an efficient **HL** reservoir as it retained 60% of the ligand during 11 days of dialysis in physiological saline. Furthermore, upon incubation with Colo 205 human colon adenocarcinoma cells for 3 days, the **HL**/HSA hydrogel did not exhibit cytotoxic activity, demonstrating that it is also an efficient ligand depot in the presence of living cells. It was observed that the percentage of **HL** release is independent of its initial concentration in the hydrogel, suggesting that HSA possesses a specific binding site for the ligand, most likely Sudlow site 2, as predicted by molecular docking. The intrinsic property of albumin to bind and transport various substances, including hydrophobic drugs, may be fine-tuned by appropriate physical/chemical hydrogel preparation procedures, providing optimal drug delivery.

## 1. Introduction

Cancer is a fatal disease, characterized by uncontrolled growth and cell division related to oncogene activation and/or tumor suppressor gene deactivation [1,2]. Regarding the risk of disease progression, early diagnosis is of essential importance for favorable clinical outcomes [3,4]. In oncology, imaging methods have become an indispensable part of the diagnostic approach [5]. The conventional imaging modalities, such as X-ray imaging, computed tomography (CT), and magnetic resonance imaging (MRI), established in cancer management, are usually followed by invasive biopsy extraction for a definite diagnosis [6]. Molecular imaging, which provides valuable insight into biological processes at cellular and sub-cellular levels, has exhibited the potential to suppress such an invasive procedure [7]. Positron emission tomography (PET) is characterized by high sensitivity, unlimited penetration depth, and competency for quantitative assessment but lacks anatomical data [8]. This issue has been overcome by combining imaging modalities into hybrid systems, such as PET/CT and PET/MRI. These sophisticated techniques have not only improved the diagnosis but were shown to be a promising tool for cancer staging and evaluation of treatment response [9,10]. Furthermore, with the growing importance of theranostics in oncology, new possibilities have been opened up for imaging, particularly multimodal imaging [11,12]. Although various theranostic platforms have been developed, their translation from animal studies to clinics remains challenging [13]. In light of this, there is still a need for the improvement of proposed methods and the involvement of other imaging techniques and probes that could be beneficial for personalized cancer care.

In terms of therapeutic procedures, conventional anticancer therapies, including surgery, radiation, and chemotherapy, are still unsurpassed, although each has numerous limitations [14,15]. Although platinum-based drugs are commonly used in chemotherapy, their therapeutic success is limited by significant side effects due to their non-selective action. While effectively destroying cancer cells, these drugs also exhibit cytotoxicity toward fast-growing healthy cells. Another critical issue of chemotherapy is DNA mutation-developed treatment resistance [16,17]. Consequently, enormous efforts are being made to design more targeted therapies, including the synthesis of new drugs and the development of novel drug delivery approaches [17]. The research focused on innovative drugs that, unlike classical chemotherapeutics, do not include DNA targeting has recently gained momentum [18]. Furthermore, drug encapsulation has shown to be advantageous over direct administration of chemotherapeutic agents, providing enhanced drug pharmacokinetic and pharmacodynamic properties, targeted delivery, and reduced chemotoxicity [19]. Among various drug vehicles, such as micelles [20], liposomes [21], and polymeric nanoparticles [22], hydrogels [23,24] have also been involved in anticancer drug studies.

Hydrogels are three-dimensional cross-linked polymeric matrices able to absorb large amounts of water while resisting dissolution [25,26]. Their unique physicochemical features and excellent biocompatibility allow for a wide range of biomedical applications, including sustained drug delivery [27,28]. The advantage of hydrogels over other vehicle formulations lies in the high water content, which makes them resemble natural tissues and thus minimizes surface tension with biological fluids [29,30]. In terms of biocompatibility, biodegradability, and non-toxicity, naturally derived hydrogels are considered more suitable for in vivo applications than those made from synthetic materials [24,31]. Various hydrogels based on biopolymers, such as chitosan [32], alginate [33], agarose [34], and gelatin [35], have been synthesized for cancer treatment. Recently, a bovine serum albumin (BSA)-based hydrogel, containing epichlorohydrin as a crosslinker, has been shown to be a suitable drug scaffold providing sustained release of doxorubicin to cancer cells [36].

Serum albumin is the most abundant and long-circulating plasma protein involved in numerous physiological functions [37,38,39,40,41,42], in addition to its essential role in the transport of endogenous and exogenous compounds. This helical-structure protein possesses multiple ligand-binding sites, allowing for the tight binding of fatty acids, bilirubin, free metal ions, and a broad spectrum of drugs [43,44,45,46]. Most recently, BSA-based hydrogels with different mechanical properties were evaluated in vitro as delivery systems for coumarin-3-carboxylic acid and warfarin, demonstrating the significance of the drug-to-protein ratio, as well as different hydrogel incubation time and gelation procedures, for tunable controlled release [47].

The vast majority of anticancer drugs are hydrophobic, making an effective delivery to the intracellular targets complicated [30]. Considering that albumin can increase the solubility of poorly water-soluble molecules [48], chemical conjugation to albumin is an alternative for overcoming this issue. Such an approach was successfully applied to avoid side reactions associated with polysorbate 80 in docetaxel clinical formulations [49,50]. Of interest for drug delivery is also albumin’s capability to prolong in vivo half-life of otherwise rapidly cleared drugs [38,48]. For example, a potent but short-acting antitumor agent, interleukin-2, was genetically fused to human serum albumin (HSA), resulting in a prolonged half-life and extended action time [51]. Similarly, tumor necrosis factor-related apoptosis-inducing ligand (TRAIL) lacks clinical application due to its poor pharmacokinetics. However, its fusion to an albumin-binding domain with high affinity extends the circulatory half-life, exerting long-lasting cytotoxic effects in vivo [52]. In addition, albumin accumulation in solid tumors due to leaky vasculature and impaired lymphatic drainage-caused enhanced permeability and retention effect is of particular convenience for targeted therapy [38,45]. Guided by these tumor-specific properties, various albumin-binding formulations have been proposed. Albumin’s uniqueness is related to its accessible cysteine, which was utilized to form the maleimide–doxorubicin and maleimide–camptothecin prodrugs. These derivatives rapidly bind to Cys-34 of endogenous albumin in situ with acid-sensitive promoted or enzymatic release at the tumor site [53,54,55,56]. A similar approach was applied in the development of platinum(II) [57,58] and platinum(IV) albumin-binding prodrugs [59], as well as albumin conjugates of organoruthenium complexes [60], in order to improve their antiproliferative activity. The most prominent are nanoparticle systems, among which Abraxane, approved for the treatment of metastatic breast cancer, non-small cell lung cancer (NSCLC), and metastatic adenocarcinoma of the pancreas, has achieved clinical success. Nanoparticle albumin-bound (nab) technology used to create nanoparticles through non-covalent interactions has been extended to several other anticancer drugs, such as rapamycin and docetaxel [61].

In this study, the binding of a highly cytotoxic modified 7,12-dihydroindolo[3,2-d][1]benzazepin-6(5H)-one (paullone) bearing the spin label 2,2,6,6-tetramethylpiperidine 1-oxyl (TEMPO) free radical (**HL**, Scheme SI, Supplementary Material) to HSA and its release were investigated by electron paramagnetic resonance (EPR) spectroscopy. **HL** belongs to a class of indolo[3,2-d]benzazepines and exhibits extraordinary antiproliferative activity in human cancer cell lines. Paullones and their metal complexes were found to inhibit cyclin-dependent kinases and glycogen synthase kinase-3 and to also target human R2 ribonucleotide reductase protein [62]. The major contribution to the biological activity of **HL** has been suggested to be attributed to the modified paullone structure at the original lactam unit, which creates a tridentate metal-binding site. Furthermore, the presence of the TEMPO radical has resulted in an increased cytotoxicity compared to the TEMPO-free counterpart, but not in all cancer cell lines. Yet, for the purpose of this work, the fact that **HL** contains a stable TEMPO radical was invaluable, as it has allowed for the use of a highly sensitive spectroscopic EPR technique to study the binding of this cytotoxic ligand to HSA, as well as its release. EPR has many advantages over typical methods for drug binding and release studies (i.e., ultracentrifugation, UV-visible spectrophotometry, fluorimetry, Raman spectroscopy), namely a nanomolar detection limit, requirement of extremely small sample volumes (30 µL), sensitivity to protein conformational changes, and experiment time efficiency. Although EPR spectroscopy has not been frequently used for this purpose, since it requires covalent modification of the ligand/drug with a stable radical (EPR-active group), it has been useful for the mechanistic study of HSA–drug association for over 20 different spin-labeled pharmaceuticals [63], as well as for controlled release studies of TEMPO-labeled coumarin-3-carboxylic acid and warfarin from BSA-based hydrogels [47]. Furthermore, the EPR spin labeling technique has also been used to investigate the binding of a mononitrosyl ruthenium complex to HSA, based on its competition with spin-labeled fatty acids for the binding sites on the protein [64], and for monitoring the drug-induced conformational changes in a triazine spin-labeled BSA [65]. The cytotoxicity of the **HL**/HSA hydrogel was assayed in the Colo 205 human colon adenocarcinoma cell line and was compared to that of the reference compound **HL**.

## 2. Materials and Methods

### 2.1. Materials

The hydrogels were prepared from HSA (≥99%, Sigma-Aldrich, St. Louis, MO, USA) in deionized water (Milli-Q, 18 MΩ·cm). The cytotoxic modified paullone ligand bearing a TEMPO free radical unit (**HL**), was synthesized by Prof. V. B. Arion’s group at the Institute of Inorganic Chemistry of the University of Vienna, as described in [62]. The characterization details are given in [62]. The spin probes TEMPO (Sigma-Aldrich, St. Louis, MO, USA), 3-carbamoyl-2,2,5,5-tetramethylpyrrolidine-1-oxyl free radical (3CP) (Sigma-Aldrich, St. Louis, MO, USA), and 5-doxyl-stearic acid (5-DS) (Sigma-Aldrich, St. Louis, MO, USA) were used for the **HL** binding site evaluation, hydrogel water diffusion, and matrix degradation studies, respectively. Physiological saline, 0.9% (*w/v*) NaCl solution (Hemofarm, Vršac, Serbia), was used as the dialysis buffer for ligand release studies. Dimethyl sulfoxide (DMSO) was purchased from Merck KGaA, Darmstadt, Germany. All cell culture reagents were obtained from Sigma-Aldrich, St. Louis, MO, USA. Human Colo 205 (chemosensitive) colon adenocarcinoma cell line was purchased from LGC Promochem, Teddington, UK.

### 2.2. The Binding and Release of **HL** from HSA

The binding and release of **HL** in HSA solutions, and corresponding thermally induced hydrogels, were measured by X-band EPR spectroscopy. The EPR spectra were acquired on a Bruker Biospin Elexsys II E540 EPR spectrometer with the following experimental parameters: microwave frequency 9.8 GHz, microwave power 10 mW, modulation amplitude 0.5 G, modulation frequency 100 kHz.

#### 2.2.1. **HL** Release from HSA Solutions

The HSA solutions were prepared by dissolving an appropriate amount of HSA in deionized water at room temperature, followed by the addition of a required volume of 1 mM **HL** in 10% DMSO/H_2_O (*v/v*), to obtain 0.5 mM **HL**/5 mM HSA (1:10) and 0.25 mM HL/5 mM HSA (1:20) solutions. The **HL**/HSA solutions were incubated for 30 min, and 30 µL samples were drawn into 1 mm diameter gas-permeable Teflon tubes (Zeus Industries Inc., Largo, FL, USA) and inserted into a quartz cuvette (inner diameter 3 mm, Wilmad-LabGlass, Vineland, NJ, USA) for EPR measurements. The EPR spectra of 0.1 mM **HL** in water and physiological saline were measured as controls. For the study of spontaneous ligand release, a 300 µL volume of the **HL**/HSA solution (1:10 and 1:20) was dialyzed against 50 mL of physiological saline at room temperature for 8 days, using the dialysis tubing with molecular weight cutoff (MWCO) of 12–14 kDa (Serva, Heidelberg, Germany). The physiological saline was replaced every 24 h. The absorbance at 280 nm of the dialyzed **HL**/HSA solution was periodically checked on a PerkinElmer LAMBDA Bio+ UV/Vis spectrophotometer to confirm that the protein concentration was constant during dialysis. The EPR spectra of the physiological saline and the **HL**/HSA solution were acquired every 24 h for 8 days.

#### 2.2.2. **HL** Release from HSA Hydrogels

The thermally induced **HL**/HSA hydrogels were prepared in a cylinder-shaped mold (2.5 mm radius base) from 1:10 and 1:20 **HL**/HSA stock solutions incubated at 75 °C for 40 min. For the study of spontaneous ligand release, the hydrogels were dialyzed against 50 mL physiological saline at room temperature for 11 days. The physiological saline was replaced every 24 h. For EPR measurements, the hydrogel was placed in the EPR tissue sample cell (Wilmad-LabGlass, Vineland, NJ, USA). Prior to placing the sample in the EPR cell, the excess physiological saline was carefully removed from the hydrogel by placing it on absorbent paper for 30 s. The EPR spectra were acquired every 24 h.

### 2.3. 5-DS/HSA and 3CP/HSA Spin-Labeled Hydrogels

To monitor the degradation of the protein matrix, a HSA hydrogel containing a spin-labeled stearic acid (5-DS) was prepared from the stock solution containing 5 mM 5-DS/5 mM HSA, using the same procedure described in Section 2.2.2. The hydrogel was dialyzed against 50 mL physiological saline at room temperature for 11 days. The rate of water/ligand diffusion from the hydrogel water pores was studied in the 0.5 mM 3CP/5 mM HSA hydrogel (prepared as described in Section 2.2.2), which was dialyzed against 50 mL physiological saline for 3 h. The EPR spectra of both hydrogels over the course of dialysis were acquired in the EPR tissue sample cell.

### 2.4. In Vitro Cell Studies on **HL** and the **HL**/HSA Hydrogel: Cell Line and Culture Conditions and MTT Assay

The cell culture plastic was obtained from Sarstedt (Nümbrecht, Germany). The human Colo 205 cells were cultured as described in our previous work using Roswell Park Memorial Institute (RPMI) 1640 medium [66]. **HL** was dissolved in DMSO using 10 mM concentration, and the DMSO content was always lower than 1% (*v/v*) in the final samples. Then the **HL** stock solution was diluted in complete culture medium, and 2-fold serial dilutions of the compound were prepared in 100 μL of the medium, horizontally. The **HL**/HSA hydrogel contained 0.5, 1, and 5 μM **HL** in a 40 μL volume. The semi-adherent colon adenocarcinoma cells were treated with Trypsin-Versene (EDTA) solution. They were adjusted to a density of 1 × 10^4^ cells in 100 μL of RPMI 1640 medium and were added to each well, with the exception of the medium control wells. The final volume of the wells containing compounds and cells was 200 μL. The plates containing the Colo 205 cells were incubated at 37 °C for 72 h; at the end of the incubation period, 20 μL of 3-(4,5-dimethylthiazol-2-yl)-2,5-diphenyl-tetrazolium bromide (MTT) solution (from a stock solution of 5 mg/mL) was added to each well. After incubation at 37 °C for 4 h, 100 μL of sodium dodecyl sulfate (SDS) solution (10% in 0.01 M HCl) was added to each well, and the plates were further incubated at 37 °C overnight. Cell growth was determined by measuring the optical density (OD) at 540/630 nm with a Multiskan EX plate reader (Thermo Labsystems, Cheshire, WA, USA). Inhibition of the cell growth (expressed as IC_50_: inhibitory concentration that reduces the growth of the cells exposed to the tested compounds by 50%) was determined from the sigmoid curve where 100 − ((OD_sample_ − OD_medium control_)/(OD_cell control_ − OD_medium control_)) × 100 values were plotted against the logarithm of compound concentrations. Curves were fitted by GraphPad Prism software (2021, Graphpad Software, San Diego, CA, USA) [67] using the sigmoidal dose–response model (comparing variable and fixed slopes). The IC_50_ values were obtained from at least three independent experiments.

### 2.5. Molecular Docking (MD)

The crystal structures of HSA (pdb ID: 2bxg) and BSA (pdb ID: 4or0) (only A chains), were cleared from heteroatoms and solvent molecules, followed by the addition of polar hydrogen atoms for MD calculations. The 3D structures of ligands were created and optimized by energy minimization using Avogadro software [68]. The ligand was treated as a flexible molecule, while the protein structure was kept rigid. Molecular docking was performed using AutoDock Tools 1.5.7 and 4.2 software [69]. Discovery Studio 2021 was used to recognize the residues involved in the binding process, as well as to visualize the 2D interaction patterns of amino acid residues in the proximity of the investigated ligands. The best structures were selected based on the docking score and number of ligand–receptor interactions.

### 2.6. EPR Imaging of the Spin-Labeled Hydrogels

The **HL**-, 5-DS-, and 3CP-containing hydrogels were placed in the EPR tissue sample cell and 2D EPR imaging was performed. The experimental parameters were: microwave frequency 9.8 GHz, gradient strength 15 G/cm, microwave power 10 mW, modulation amplitude 1 G, modulation frequency 100 kHz, sampling time 0.03 s, field-of-view 13 mm, pixel size 0.1 mm. The images were processed with Bruker Xepr software (*Xepr 2.6b.84*, Bruker BioSpin GmbH, Rheinstetten, Germany).

## 3. Results and Discussion

### 3.1. **HL** Binding to HSA

**Figure 1:**
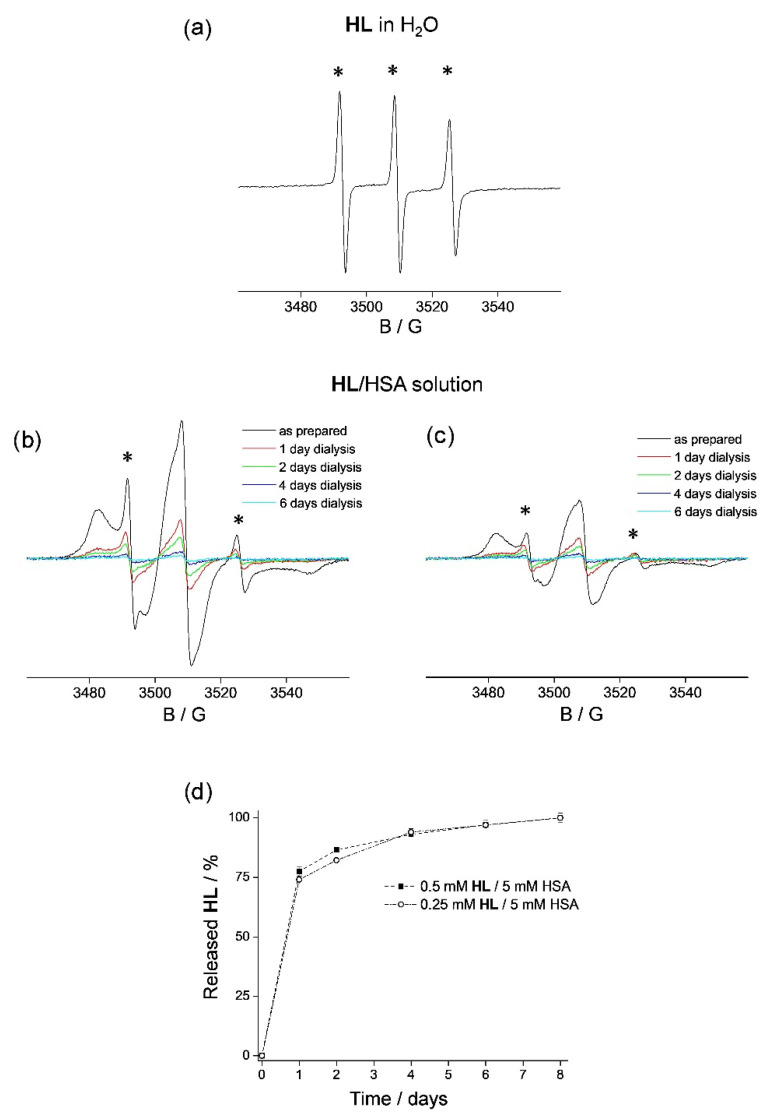
The X-band EPR spectra of (**a**) 0.1 mM **HL** in water, (**b**) 0.5 mM **HL**/5 mM HSA solution, and (**c**) 0.25 mM **HL**/5 mM HSA solution, as prepared (black) and after 1 day (red), 2 days (green), 4 days (blue), and 6 days (cyan) of dialysis in physiological saline at room temperature. The spectra in (**b**,**c**) are shown on the same scale; the y-scale for the spectrum in (**a**) is reduced by 2x. The signals that arise from **HL** in water (not bound to HSA) in (**b**,**c**) are marked with an asterisk. (**d**) The rate of spontaneous **HL** release from solutions containing 0.5 mM **HL**/5 mM HSA (squares) and 0.25 mM **HL**/5 mM HSA (open circles) in physiological saline.

**HL** is suggested to be in its neutral form at physiological pH due to the determined p*K*_a_ values of analogous compounds [70]. The EPR spectra of **HL** in water (Figure 1a) and in the physiological saline are identical, exhibiting isotropic three-line signals that arise from the TEMPO label, and typical for free-tumbling nitroxides [71,72]. The EPR spectra of **HL** incubated with a solution of HSA for 30 min at room temperature, for the **HL**:HSA molar ratios 1:10 (Figure 1b, black line) and 1:20 (Figure 1c, black line), display spectral line broadening, indicating restricted motion of the spin-labeled ligand **HL** [71,72,73], i.e., **HL** binding to HSA. Besides the presence of the protein-bound **HL**, the spectra also display a minor contribution from **HL** in water (marked with *), which is not bound to HSA. From the height of the high-field EPR line of the unbound **HL**, it was possible to estimate that the 1:10 (molar ratio) **HL**/HSA solution contains 90% protein-bound **HL** and 10% unbound **HL**, whereas the 1:20 solution contains 94% bound and 6% unbound ligand. Based on these binding data, assuming the 1:1 stoichiometry for the ligand–HSA adduct, a log*K* ~ 3.4 ± 0.2 formation constant can be estimated, which suggests a moderate binding. To determine the spontaneous in vitro release rate of **HL** from HSA in both solutions, the solutions were dialyzed against physiological saline at room temperature. Specifically, 300 µL of both **HL**/HSA solutions were dialyzed against 50 mL of physiological saline for 8 days, and the EPR spectra were acquired every 24 h. The amount of **HL** displaced from the protein was determined by spin quantification (double integration) of the signal from the **HL**/HSA solutions. In both cases, it was observed that ~75% of **HL** is released from the protein after 1 day (Figure 1b,c, red lines). The presence of **HL** in physiological saline, released from the **HL**/HSA solutions during dialysis, was also confirmed by EPR spectroscopy (Figure S1, Supplementary Material). After 2 days, the rate of **HL** release was greatly reduced, leading to ~85% total ligand displacement (Figure 1b,c, green lines); 94% total ligand displacement was found after 4 days (Figure 1b,c, blue lines), and 97% total ligand displacement was found after 6 days (Figure 1b,c, cyan lines). Finally, after 8 days, the presence of **HL** was completely undetected in the solutions containing HSA. These observations show that HSA releases the same percentage of **HL**, irrespective of the initial concentration of the ligand (Figure 1d), indicating that the protein has a specific binding site for **HL**. Moreover, it can be concluded that it may be possible to adjust the absolute amount of the delivered compound by selecting the appropriate initial concentration in the HSA solution, which is essential for controlled drug delivery.

It is important to note here that the observed in vitro release rate should be expected to be much faster in vivo due to the reactions of HSA with enzymes and endogenous metabolites. Therefore, additional experiments were carried out using the HSA hydrogel in place of the solution to investigate if the hydrogel would be a more suitable **HL** depot that could potentially increase the availability of the cytotoxic ligand in vivo.

### 3.2. **HL** Binding to HSA Hydrogel Matrix

**Figure 2:**
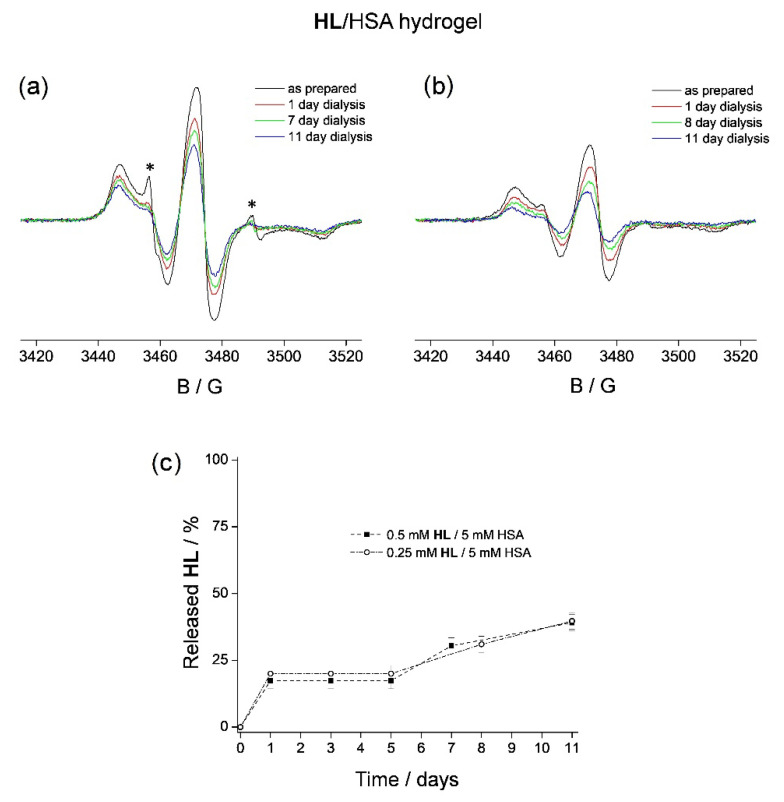
The X-band EPR spectra of (**a**) 0.5 mM **HL**/5 mM HSA hydrogel and (**b**) 0.25 mM **HL**/5 mM HSA hydrogel, as prepared (black) and after 1 day (red), 7 (or 8) days (green), and 11 days (blue) of dialysis in physiological saline at room temperature. The spectra are shown on the same scale. The signals that arise from **HL** in water (not bound to HSA) in (**a**) are marked with an asterisk. (**c**) The rate of spontaneous **HL** release from hydrogels containing 0.5 mM **HL**/5 mM HSA (squares) and 0.25 mM **HL**/5 mM HSA (open circles) in physiological saline.

The **HL**/HSA hydrogels were prepared by heating the 1:10 and 1:20 **HL**/HSA stock solutions for 40 min at 75 °C [74]. It should be highlighted that increased temperature does not affect the EPR spectrum of **HL**. The spectra of the thermally induced **HL**/HSA hydrogels (Figure 2a,b) show the presence of the bound **HL**, as observed in the HSA solution (Figure 1b,c). However, the contribution of the unbound **HL** in the 1:10 **HL**/HSA gel (located in its water pores) was smaller compared to that in the stock solution, and it was undetected in the 1:20 **HL**/HSA gel. Most likely, this is the consequence of HSA conformational change during the heating process, in which the protein becomes more accessible to the ligand, prior to the formation of the β-sheet denatured form [75,76]. This is also in agreement with the observed broadening of the EPR spectrum (65.6 G in Figure 2, compared to 64.8 G in Figure 1), indicating stronger binding of **HL** to HSA in the hydrogel compared to that in the solution [77]. To determine the spontaneous in vitro release rate of **HL** from the HSA hydrogels, both were dialyzed against 50 mL of physiological saline at room temperature, for 11 days, and the EPR spectra were acquired every 24 h. After the first 24 h, both hydrogels released ~20% **HL** (Figure 2a,b, red lines). The amount of the displaced **HL** was determined, as previously, by spin quantification. Following this, there was no further **HL** release from the 1:10 HL/HSA gel until day 7, when an additional 10% **HL** was released from the HSA matrix (Figure 2a, green line). The presence of **HL** in the hydrogel pores was confirmed by the appearance of the EPR signal from free **HL** (marked with *). Subsequent measurements showed that there is an additional 10% ligand displacement (40% total) from this hydrogel after 11 days (Figure 2a, blue line). The 1:20 **HL**/HSA gel showed a similar rate of ligand release, namely 30% and 40% after 8 and 11 days, respectively (Figure 2b, green and blue lines). It is apparent that the hydrogels were able to retain a significant amount of **HL** while stored in the physiological saline for 8 days (Figure 2c and Table 1), during which the corresponding **HL**/HSA solutions released 100% **HL** (Figure 1d). This confirms that the hydrogel is more applicable as a potential **HL** depot than the HSA solution.

The observed ligand displacement after 7 days from the 1:10 **HL**/HSA hydrogel protein matrix (and 8 days from 1:20) into the hydrogel water pores is most likely due to the weakening of the **HL**–HSA interaction, suggesting that at this point, the hydrogel matrix has started to degrade. Therefore, further investigations of the HSA hydrogel, focused separately on the protein matrix and on the water phase during dialysis, were performed. Specifically, two additional types of HSA hydrogels were prepared, one containing a spin-labeled fatty acid, 5-doxylstearic acid (5-DS), to study the matrix and the other containing the spin probe 3-carbamoyl-proxyl (3CP) to study the water phase.

### 3.3. HSA Hydrogel Matrix Degradation

Hydrogel degradation upon storage in water solutions (buffers, physiological saline), especially at room temperature, is expected. This process may be studied at the molecular level by evaluating the interactions between the protein and the ligand. Therefore, in order to monitor the degradation of the HSA matrix, which may have been the reason for the observed **HL** release from the 1:10 **HL**/HSA hydrogel after 7 days of dialysis, a different type of a spin-labeled HSA hydrogel was prepared. For this purpose, a HSA hydrogel containing spin-labeled stearic acid (5-DS) was used. The idea was to investigate hydrogel degradation using the same EPR protocol that was used for **HL** binding and release (hence the need for the spin-label). However, in this case, a fatty acid that strongly binds, and therefore is not expected to readily dissociate from the HSA hydrogel in physiological saline during the experimental timeline. It is well known that HSA (and BSA as well) represents the main carrier for fatty acids in blood plasma and contains at least seven binding sites for medium- and long-chain fatty acids [44,78,79,80]. In line with this, it has also been shown that up to six equivalents of the spin-labeled derivatives of stearic acid display strong binding to HSA [81,82,83,84].

**Figure 3:**
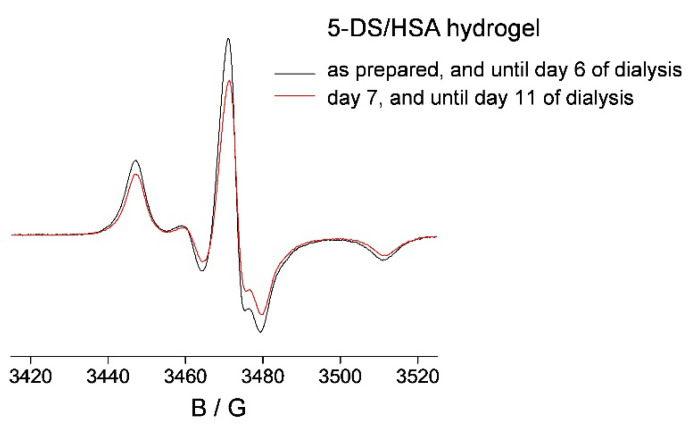
The X-band EPR spectra of 5 mM 5-DS/5 mM HSA hydrogel, as prepared, up to 6 days of dialysis (black), and after 7 (until 11) days of dialysis (red), in physiological saline at room temperature.

The 5-DS/HSA hydrogel (1:1 molar ratio) stored in physiological saline was monitored by EPR over a period of 11 days. No change in the EPR spectrum of the 5-DS/HSA hydrogel was observed up to 6 days of dialysis (Figure 3, black line), indicating no displacement of 5-DS during this time period, unlike the observed 20% **HL** release from the **HL**/HSA gel after only 1 day (Figure 2). However, after 7 days, a total of 20% of 5-DS was released (Figure 3, red line). Further ligand release was not observed during the experimental timeline. This shows that both hydrogels, **HL**/HSA and 5-DS/HSA, liberate the corresponding ligand after 7 days of dialysis, indicating the onset of the hydrogel matrix degradation at this time.

The ligand release after 7 days is likely caused by the disturbance of the non-covalent interactions between the ligand and the protein, most probably as the result of the uptake of physiological saline during dialysis, which has an ionic strength of 154 mM. This observation is quite useful for the prediction of the hydrogel function in vivo, as the extracellular ionic strength may be higher, and faster release of **HL** from the hydrogel could be expected. In addition, hydrogel degradation caused by enzymes and endogenous metabolites would certainly result in an even higher delivery rate of **HL** to cells. This highlights the experimental limitations of the in vitro studies and the inevitable problem when translating knowledge obtained from delivery vehicle characterization in a model system to in vivo applications.

On a side note, the solution of 5-DS/HSA was not able to retain the spin-labeled fatty acid as the corresponding hydrogel, releasing 95% after 7 days after dialysis (Figure S2, Supplementary Material), confirming the conclusion made for **HL**/HSA that the hydrogel is a more suitable ligand depot than the solution for other compounds as well.

### 3.4. **HL** Diffusion from HSA Hydrogel Water Pores

**Figure 4:**
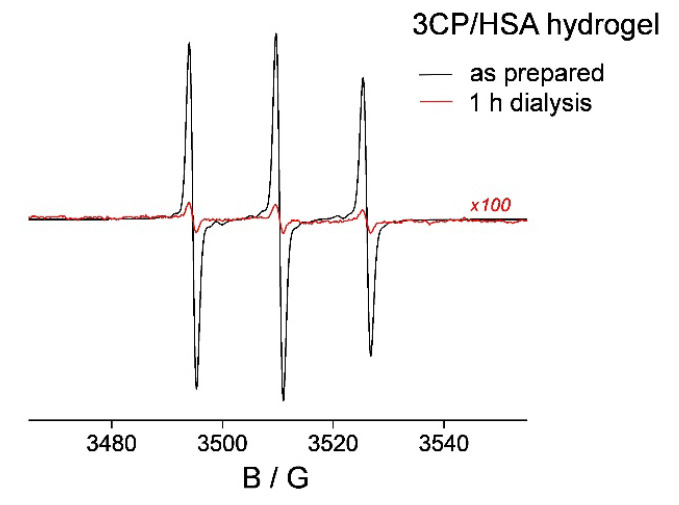
The X-band EPR spectra of the 0.5 mM 3CP/5 mM HSA hydrogel, as prepared (black line) and after dialysis in physiological saline for 1 h at room temperature (red line). The y-scale for the spectrum shown in red is magnified by 100× for clarity. The calculated rotational correlation times of 3CP are 0.13 ns for both spectra, corresponding to the hydrogel water content of 2.2 mg H_2_O/1 mg HSA.

The results thus far showed that upon **HL**/HSA hydrogel preparation in the molar ratio 1:10, most of the **HL** is bound to HSA. The amount of **HL** not bound to HSA, and dissolved in the hydrogel water pores, was found to diffuse from the hydrogel into physiological saline after 24 h (Figure 2). Then, after 7 days, additional **HL** was released from the protein matrix into the water pores of the hydrogel and subsequently diffused from the water pores into the physiological saline. The process that governs the rate of **HL** diffusion from the hydrogel pores is largely determined by the rate of water exchange with physiological saline (osmosis and Na^+^Cl^−^ ion diffusion). Namely, it has been shown that for this type of hydrogel, which was synthesized together with the drug, the water uptake during the swelling process leads to the release of the entrapped molecule [85]. Since the diffusion kinetics of the solute throughout the gel matrix depend on the relative size of the drug compared to that of the water pore [86,87,88], the amount of water within hydrogel pores is crucial for controlled drug delivery. Therefore, in order to gain more information about the rate of **HL** diffusion from the hydrogel water pores, a third type of spin-labeled HSA hydrogel was prepared. The idea was to take advantage of the recently reported methodology for hydrogel water content determination using the spin probe 3CP [74]. This EPR-active molecule was shown to be entirely dissolved in the water phase of the hydrogel, not interacting with the protein matrix. The 3CP/HSA hydrogel was prepared in the same ligand-to-protein molar ratio as the 1:10 **HL**/HSA gel and investigated using the same protocol. As determined previously [74], 3CP does not bind to HSA, and the 3CP/HSA hydrogel displays an isotropic three-line EPR signal (Figure 4, black line). Upon 3CP/HSA hydrogel dialysis in physiological saline at room temperature, it was determined that already after 1 h, 96% of 3CP had diffused out (Figure 4, red line; note that the *y*-axis is magnified by 100× compared to the black line). This result shows that the rate of water diffusion in and out of this particular HSA hydrogel is a relatively fast process compared to the rate of the ligand release from the protein (Figure 2). Therefore, for sustained drug delivery, it is obvious that the limiting factor is the rate of drug release from the hydrogel matrix. In this context, HSA is definitively the most appropriate biocompatible drug depot known to date, as it can bind hundreds of drugs with relatively high affinity.

### 3.5. In Vitro Cytotoxicity of **HL** and **HL**/HSA Hydrogel

The cytotoxic activity of **HL** and the **HL**/HSA hydrogel was measured by the MTT assay in Colo 205 human colon adenocarcinoma cells, using 72 h incubation time. In the case of the hydrogel, the assay was performed via its direct contact with the cells. The standalone cytotoxicity of **HL** was already determined in a series of human cancer cell lines (A549, CH1, SW480, N87, SK-Mel 28, T47D) in our previous work [62], and IC_50_ values varied between 0.02 and 0.27 μM, using 96 h exposure time, showing its high activity. In the present work, measurements were performed with the aim of investigating if the **HL**/HSA hydrogel displays cytotoxicity during 72 h, in order to determine if it releases the active ligand during this incubation time with the Colo 205 cells. For **HL** alone, a value of IC_50_ = 0.090 ± 0.001 μM was obtained; thus, the compound is strongly cytotoxic against the tested cell line. Note that the IC_50_ values for doxorubicin, oxaliplatin, 5-fluorouracil, etoposide, and regorafenib in Colo 205 cells are (3.3 ± 0.2) µM [89], (2.60 ± 0.52) µM [90], 3.2 µM [91], (1.61 ± 0.02) µM [92], and 3.269 µM [93]. However, no measurable activity was found for the **HL**/HSA hydrogel during the 72 h exposure time at the applied 0.5, 1.0, and 5.0 μM **HL** concentrations. Based on these data, it can be concluded that the hydrogel could act as an efficient reservoir for **HL**, as no compound was liberated during the tested period.

### 3.6. Molecular Docking of **HL** and TEMPO to HSA

**Figure 5:**
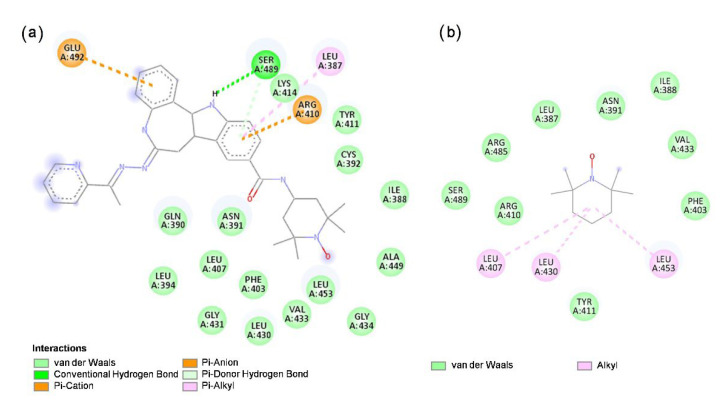
Two-dimensional ligand interaction patterns of amino acid residues in the proximity of (**a**) **HL** and (**b**) TEMPO, at Sudlow site 2 of HSA (pdb ID: 2bxg).

Molecular docking of **HL** and its structural segment, TEMPO, was performed within HSA subdomains IIA and IIIA, which contain the two principal drug-binding sites, Sudlow sites 1 and 2, respectively [44,94]. The results showed that both **HL** and TEMPO are accommodated within the hydrophobic cavity of the subdomain IIIA in HSA (Figure S3, Supplementary Material, also showing the calculations performed for ligand binding to BSA). This binding site corresponds to the drug-binding Sudlow site 2 [44], as observed from the calculated positions of the amino acid residues in the proximity of the investigated ligands (Figure 5). The docking of the ligands within Sudlow site 1 was not successful. The TEMPO structural segment of **HL** bound to HSA (Figure 5a) was found to be located in the protein hydrophobic cavity, which is in agreement with the calculated position for TEMPO only (Figure 5b). In addition to the hydrophobic interactions which may contribute to its binding, **HL** displays hydrogen bonding to Ser489 through the nitrogen atom of the indole ring which acts as a proton donor to Ser489, whereas in the case of TEMPO, no hydrogen bonding is observed. This may be the reason for the relatively higher calculated binding affinity of **HL** to HSA (and also BSA), compared to TEMPO (Table 2), and indicates that **HL** most likely binds to HSA via the paullone backbone.

**Figure 6:**
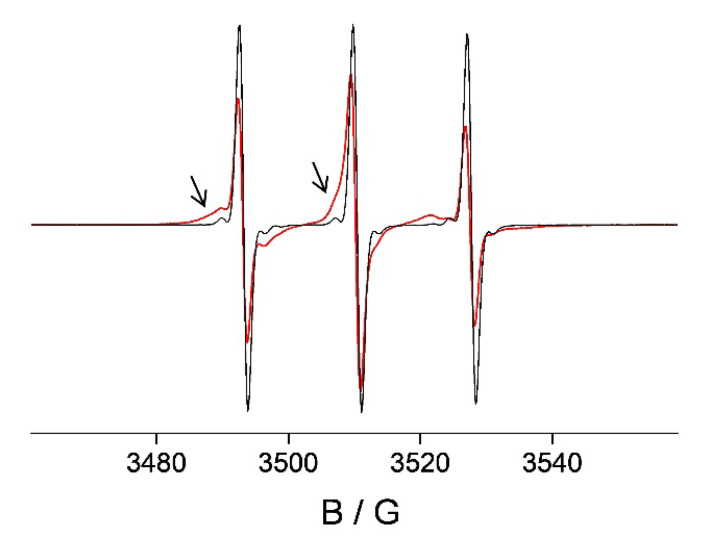
EPR spectra of 0.5 mM TEMPO in water (black) and in 5 mM HSA solution (red). The contribution from the immobilized spin probe is marked with arrows.

The different binding affinities of **HL** and TEMPO for HSA predicted by the MD calculations were also experimentally confirmed by EPR. Unlike **HL**, which shows strong binding to HSA (Figure 1b,c), TEMPO is only weakly immobilized when incubated with HSA (Figure 6), corroborating that the binding mode of **HL** to HSA does not occur via the free radical moiety.

### 3.7. EPR Imaging of Spin-Labeled Hydrogels

**Figure 7:**
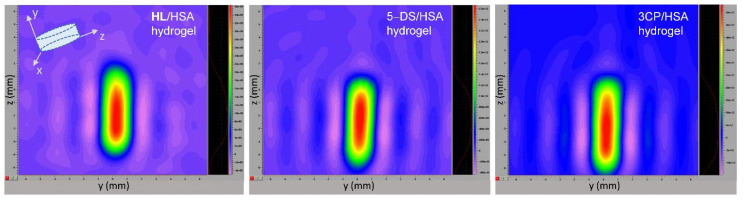
Two-dimensional *yz*-plane EPR images of 0.5 mM **HL**/5 mM HSA, 5 mM 5-DS/5 mM HSA, and 0.5 mM 3CP/5 mM HSA hydrogels (left to right). The images were processed with Bruker Xepr software.

The three types of spin-labeled HSA hydrogels investigated in this study were visualized using EPR imaging (EPRI) (Figure 7). The 2D images of the hydrogel cross-section (here specifically the yz-plane) provide confirmation that all spin-labeled compounds are homogeneously distributed throughout the entire hydrogel volume. It is important to emphasize that the EPR images were possible to be obtained not only from a narrow-line isotropic EPR signal, such as that of 3CP dissolved in the hydrogel water pores (Figure 4), but also from anisotropic signals that arise from protein-bound spin labels, as in the cases of **HL** and 5-DS (Figure 2 and Figure 3, respectively). This observation may be valuable for future biomedical applications of in vivo EPRI.

## 4. Conclusions

The binding and release of a cytotoxic paullone–TEMPO drug candidate (**HL**) from HSA were investigated in vitro, in solution, and in a thermally induced hydrogel, using EPR spectroscopy. The results showed that **HL** binds to HSA; specifically, for the **HL**:HSA = 1:10 molar ratio, 90% of **HL** is protein-bound in the solution, and 97% in the corresponding hydrogel, whereas for the 1:20 molar ratio, 94% **HL** is bound in solution, and 100% in the hydrogel. The MD calculations indicated that **HL** most likely binds to HSA via the paullone backbone, within the drug-binding Sudlow site 2. The 2D EPR imaging showed a homogeneous distribution of **HL** throughout the entire hydrogel volume.

Upon dialysis of the **HL**/HSA solutions in physiological saline at room temperature, most of the bound ligand (75%) is released during the first 24 h, after which the rate is significantly reduced, resulting in 100% ligand release after 8 days. In contrast, the corresponding hydrogels were able to retain a considerable amount (60%) of **HL** during 11 days of dialysis, showing that the hydrogel is a more suitable **HL** reservoir than the HSA solution. Furthermore, it was observed that the percentage of **HL** release is independent of its initial concentration, in both the solution and the hydrogel, indicating that the protein contains a specific binding site for **HL**. Therefore, it may be possible to adjust the absolute amount of the delivered ligand by selecting its appropriate initial concentration in the HSA stock solution, which is essential for controlled drug delivery.

The **HL**/HSA hydrogels were shown to release 20% of **HL** from the protein matrix during the first 24 h, an additional 10% after 7–8 days, and a further 10% after 11 days. The presence of the liberated **HL** in the water pores of the 1:10 **HL**/HSA hydrogel was evident from the EPR spectrum measured on day 7. This confirms that the matrix first releases the ligand into the pores, before its diffusion from the hydrogel into physiological saline. To separately investigate these processes, two additional hydrogels were prepared, both spin-labeled, allowing for the application of the EPR protocol used for **HL**/HSA analysis. The first type of hydrogel utilized for matrix degradation evaluation was spin-labeled with a fatty acid derivative 5-DS. The second, used to study the rate of water diffusion from the hydrogel pores, contained the water-soluble spin probe 3CP, which has been previously shown to be located only in the hydrogel water pores and unable to bind to HSA [74]. The results showed that **HL** displacement after 7–8 and 11 days is the consequence of hydrogel degradation, attributed to the weakening of the **HL**–HSA interaction upon physiological saline uptake. The 3CP/HSA experiments showed that 96% of 3CP diffuses out within 1 h. Therefore, it is obvious that the total (determined in this work) release rate of **HL** from the hydrogel is limited by the rate of ligand dissociation from the protein matrix. In this context, HSA represents the most appropriate biocompatible drug-depot/controlled-release vehicle known to date, as it can bind hundreds of drugs with relatively high affinity. Certainly, future studies should be focused on designing HSA-mimetics with tunable drug-binding and -releasing properties.

The successful use of spin-labeled albumin hydrogels for EPR characterization of ligand binding and release, protein matrix degradation, and the rate of ligand diffusion from the hydrogel water pores was demonstrated in this work. Together with our previous findings showing their applicability for cell viability assessment [95] and hydrogel water content determination [74], the role of state-of-the-art EPR for biomedical applications is emphasized, particularly in cancer research. Finally, the fact that the **HL**/HSA hydrogel did not exhibit cytotoxic activity in the Colo 205 human cancer cell line during the 72 h exposure time indicates that it acts as an efficient reservoir for the active ligand, even in the presence of living cells. This reveals yet another potential use of spin-labeled albumin hydrogels for continuous monitoring of drug-treatment response. For this purpose, the hydrogel would be labeled with an oximetric spin probe and loaded with an anticancer drug that is not EPR-active. This experimental approach is based on the principles of EPR oximetry, which has been successfully employed to measure pO_2_ in tissues in vivo and has found its application in cancer imaging, allowing the hypoxic tumor tissues to be distinguished from the healthy ones [96,97,98]. Oximetry-based spectroscopy and imaging could be performed prior to, during, and after the application of the drug-loaded spin-labeled hydrogel, providing information about the oxidative status of the treated cells or tissues and in turn reporting on treatment efficacy in real time. The multirole hydrogel, with integrated therapeutic and diagnostic functions, may also be convenient in anticancer drug screening in preclinical trials.

## Figures and Tables

**Table 1:** In vitro **HL** binding and release from the 5 mM HSA solution and the corresponding thermally induced hydrogel.

Sample	Day	Observation	
		*1:10 molar ratio*	*1:20 molar ratio*
***HL*** */HSA solution*			
as prepared		⮚ 90% **HL** bound to HSA, 10% **HL** unbound to HSA dissolved in water	⮚ 94% **HL** bound to HSA, 6% **HL** unbound to HSA dissolved in water
after dialysis in physiological saline	1	⮚ 75% **HL** release (total)	⮚ 75% **HL** release (total)
	8	⮚ 100% **HL** release (total)	⮚ 100% **HL** release (total)
***HL*** */HSA hydrogel*			
as prepared		⮚ 97% **HL** bound to HSA, 3% **HL** unbound to HSA dissolved in hydrogel water pores	⮚ 100% **HL** bound to HSA
after dialysis in physiological saline	1	⮚ 20% **HL** release	⮚ 20% **HL** release
	7	⮚ +10% **HL** release (30% total)	⮚ No change
	8	⮚ No change	⮚ +10% **HL** release (30% total)
	11	⮚ +10% **HL** release (40% total)	⮚ +10% **HL** release (40% total)

**Table 2:** The calculated binding affinities of **HL** and TEMPO for HSA (pdb ID: 2bxg) and BSA (pdb ID: 4or0).

Ligand	Calculated Binding Affinity (kJ/mol)	
	HSA	BSA
**HL**	−33.89	−30.54
TEMPO	−25.52	−17.57

## Data Availability

All data is contained in this article and its supporting information.

## Supplementary Materials

The following supporting information can be downloaded at: https://www.mdpi.com/article/10.3390/pharmaceutics14061174/s1, Scheme SI: The structure of 7,12-dihydroindolo[3,2-d][1]benzazepin-6(5H)-one bearing the spin label 2,2,6,6-tetramethylpiperidine 1-oxyl (TEMPO) free radical (**HL**); Figure S1: The X-band EPR spectra of **HL** in 50 mL physiological saline after 24 h dialysis at room temperature of (a) 300 µL 0.5 mM **HL**/5 mM HSA solution and (b) 300 µL 0.25 mM HL/5 mM HSA solution; Figure S2: (a) The X-band EPR spectra of 5 mM 5-DS/5 mM HSA solution as prepared (black) and after 3 days (red), 4 days (green), 5 days (blue), and 7 days (cyan) of dialysis in 50 mL physiological saline at room temperature. (b) The rate of spontaneous 5-DS release from the 300 µL 5 mM 5-DS/5 mM HSA solution during dialysis; Figure S3: The calculated location of **HL** and TEMPO within Sudlow 2 site of HSA, pdb ID: 2bxg, (a,b), and BSA, pdb ID: 4or0, (c,d), respectively. The superimposed protein structures are represented in grey, and ligand structures are represented in purple.
