# Supplementary material for: The Release of a Highly Cytotoxic Paullone Bearing a TEMPO Free Radical from the HSA Hydrogel: An EPR Spectroscopic Characterization
Source: Pharmaceutics. 2022 May 30;14(6):1174. doi: 10.3390/pharmaceutics14061174 (PMC9227768; doi:10.3390/pharmaceutics14061174)
Supplement: Supplementary file 1 [file pharmaceutics-14-01174-s001.zip › pharmaceutics-1732951-supplementary.pdf]

Supplementary Material

for

The Release of a Highly Cytotoxic Paullone Bearing a TEMPO Free Radical from the HSA Hydrogel: An EPR Spectroscopic Characterization

Ana Vesković[1], Đura Nakarada[1], Olga Vasiljević[1], Anatolie Dobrov[2], Gabriella Spengler[3,4], Éva A. Enyedy[3], Vladimir B. Arion[2], Ana Popović Bijelić[1]*

[1]*Faculty of Physical Chemistry, University of Belgrade, Studentski trg 12-16, 11158 Belgrade, Serbia*

[2]*Institute of Inorganic Chemistry, University of Vienna, Währinger Strasse 42, A-1090 Vienna, Austria*

[3]*MTA-SZTE Lendület Functional Metal Complexes Research Group, Department of Inorganic and Analytical Chemistry, University of Szeged, Dóm tér 7, H-6720 Szeged, Hungary*

[4]*Department of Medical Microbiology, Albert Szent-Györgyi Health Center and Albert Szent-Györgyi Medical School, University of Szeged, Semmelweis u. 6, H-6725 Szeged, Hungary*

[*]Corresponding author: Ana Popović Bijelić, ana@ffh.bg.ac.rs; EPR Laboratory, Faculty of Physical Chemistry, University of Belgrade, Studentski trg 12-16, 11158 Belgrade, Serbia; Phone/Fax. +381(0)112187133.

List of Figures:

Scheme SI. The structure of 7,12-dihydroindolo[3,2-d][1]benzazepin-6(5H)-one bearing the spin label 2,2,6,6-tetramethylpiperidine 1-oxyl (TEMPO) free-radical (**HL**).

Figure S1. The X-band EPR spectra of **HL** in 50 mL physiological saline after 24 h dialysis at room temperature of (**a**) 300 µL 0.5 mM **HL** / 5 mM HSA solution, and (**b**) 300 µL 0.25 mM **HL** / 5 mM HSA solution.

Figure S2. (**a**) The X-band EPR spectra of 5 mM 5-DS / 5 mM HSA solution as prepared (black), after 3 days (red), 4 days (green), 5 days (blue), and 7 days (cyan) of dialysis in 50 mL physiological saline at room temperature. (**b**) The rate of spontaneous 5-DS release from the 300 µL 5 mM 5-DS / 5 mM HSA solution during dialysis.

Figure S3. The calculated location of **HL** and TEMPO within Sudlow 2 site of HSA, pdb ID: 2bxg, (**a** and **b**), and BSA, pdb ID: 4or0, (**c** and **d**), respectively. The superimposed protein structures are represented in grey, and ligand structures are represented in purple.

1. Structure of the cytotoxic ligand HL

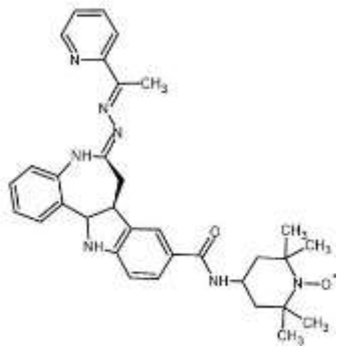

Scheme I. The structure of 7,12-dihydroindolo[3,2-d][1]benzazepin-6(5H)-one bearing the spin label 2,2,6,6-tetramethylpiperidine 1-oxyl (TEMPO) free-radical (**HL**).

2. HL release from the HSA solution and hydrogel during dialysis

The presence of the cytotoxic paullone ligand bearing a TEMPO free-radical group (**HL**) in physiological saline, released from the **HL**/HSA solutions and hydrogels during the course of dialysis was monitored by EPR spectroscopy. Representative spectra are shown for **HL** in 50 mL physiological saline after 24 h dialysis of 300 μL 5 mM HSA solutions containing 0.5 or 0.25 mM **HL,** Figure S1.

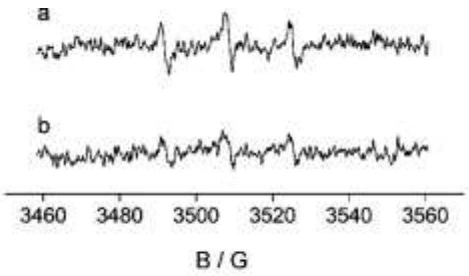

Figure S1. The X-band EPR spectra of **HL** in 50 mL physiological saline after 24 h dialysis at room temperature of (**a**) 300 μL 0.5 mM **HL** / 5 mM HSA solution, and (**b**) 300 μL 0.25 mM **HL** / 5 mM HSA solution.

3. 5-DS release from the HSA solution during dialysis

The dialysis of a 300 μL 5 mM 5-doxyl stearic acid (5-DS) / 5 mM HSA solution in 50 mL physiological saline at room temperature for 7 days was performed as a control experiment for that of the corresponding hydrogel. It was determined that the solution was not able to retain the spin-labeled fatty acid as the hydrogel, releasing 95 % 5-DS after 7 days, Figure S2.

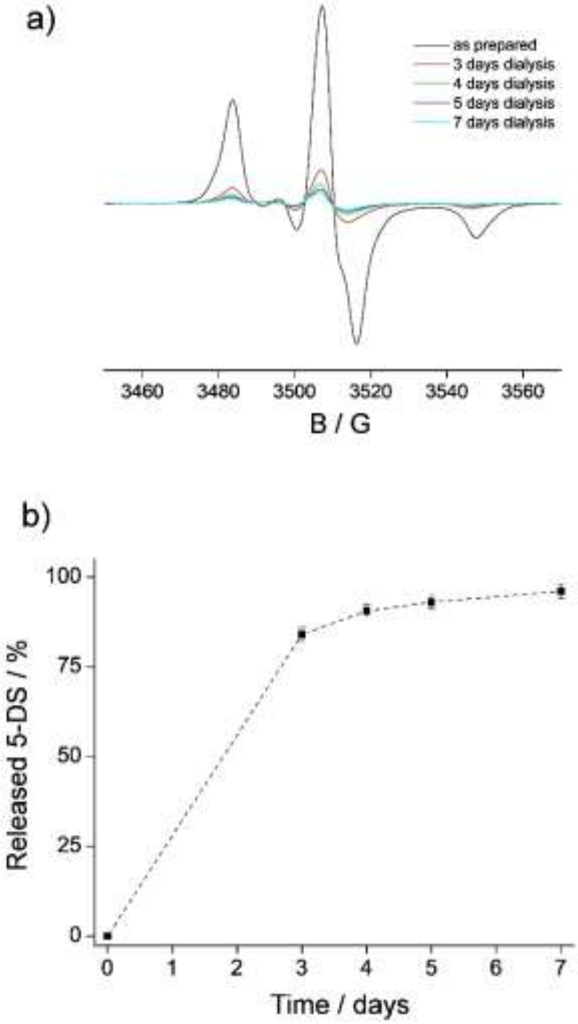

Figure S2. (**a**) The X-band EPR spectra of 5 mM 5-DS / 5 mM HSA solution as prepared (black), after 3 days (red), 4 days (green), 5 days (blue), and 7 days (cyan) of dialysis in 50 mL physiological saline at room temperature. (**b**) The rate of spontaneous 5-DS release from the 300 μL 5 mM 5-DS / 5 mM HSA solution during dialysis.

4. Molecular docking of HL and TEMPO to HSA and BSA

The MD calculations showed that both, **HL** and its structural segment, TEMPO, are accommodated within the hydrophobic cavity of the subdomain IIIA (which contains the drug-binding, Sudlow 2 site) in HSA, as well as in BSA, Figure S3.

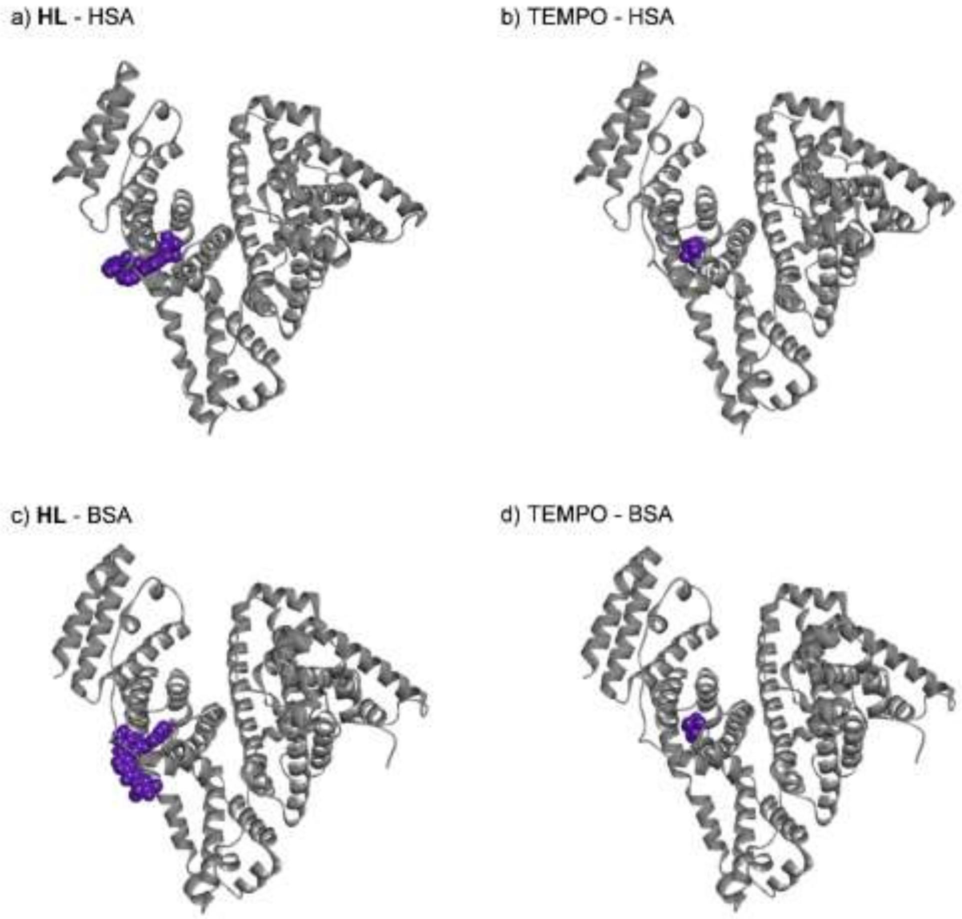

Figure S3. The calculated location of **HL** and TEMPO within Sudlow 2 site of HSA, pdb ID: 2bxg, (**a** and **b**), and BSA, pdb ID: 4or0, (**c** and **d**), respectively. The superimposed protein structures are represented in grey, and ligand structures are represented in purple.
